# Supplementary material for: Oral Administration of Okara Soybean By-Product Attenuates Cognitive Impairment in a Mouse Model of Accelerated Aging
Source: Nutrients. 2019 Dec 3;11(12):2939. doi: 10.3390/nu11122939 (PMC6950093; doi:10.3390/nu11122939)
Supplement: Supplementary file 1 [file nutrients-11-02939-s001.pdf]

Supplement 1. Sequences of the primers used in qPCR.

Genes		Nucleotide
bdnf	Forward	5'-ATCCTGTCCAAACTAAGGCTCG-3'
	Reverse	5'-ACCTCTTTAGCATAGTAGTCCGC-3'
nt3	Forward	5'-CAGGCTGCTGTAACGATGAA-3'
	Reverse	5'-AATGCTTTCTCCGCTCTGAA-3'
il10	Forward	5'-GTGTGGACCGAGGGGCTTTTACTTC-3'
	Reverce	5'-GCTTCAGTGGGGCACAGTACATCTC-3'
tnfa	Forward	5’-CCGCTGCATATCGTCCTGTG-3’
	Reverse	5’-AGTGGATGGATGGTCCTATTACA-3’
il1b	Forward	5’-GCAAGCAACAACCCGATACC-3’
	Reverse	5’-CCCTGTTTGCCGCATATAGAA-3’
inos	Forward	5'-TCCTGCCGATGTCGCTATC-3'
	Reverse	5'-CAAGTTCCGGTGTGACTCGTG-3'
ChAT	Forward	5'-CAAGCCGAGAATGCTGAGTTCATG-3'
	Reverse	5'-GCAAGGGATGATTTCCTGCCAG-3'
AChe	Forward	5'-AGGATGGGAGGTACTCGAATC-3'
	Reverse	5'-AGGCGTCCTTCCTTATATGCTA-3'
β-actin	Forward	5'-CACTATTGGCAACGACAAGCGGTTC-3'
	Reverse	5'-ACTTGCGGTGCACGATGGAG-3'
